# Ontario College

**Published:** 1896-04

**Authors:** 


### THE ONTARIO VETERINARY COLLEGE.

DR. EDMUND E. KING, of Toronto, gave an excellent address to the students in the lecture hall of the Ontario Veterinary College, on Thursday evening, February 20th, on the new photographic process called the "Cathodic Rays." His explanation of the method, which he demonstrated by the use of apparatus, was exceedingly clear, and warmly appreciated by the students. He said that it was more correctly called the "X-rays," and that it differed from photography in representing not the image or figure itself, but its shadow; that bones within the body of a man or an animal could be readily examined by this method; and that it was without doubt destined to be of inestimable benefit to medical science in the near future. Any new investigations or discoveries that benefit human medicine will certainly prove both interesting and beneficial to the sister science.

Dr. King's lecture was listened to with marked attention, and at its close a unanimous vote of thanks was tendered him by the students.

